# Quality-adjusted tooth years (QATY) as an outcome measure of periodontal treatment

**DOI:** 10.1186/1471-2458-14-S1-P10

**Published:** 2014-01-29

**Authors:** Tuti Mohd-Dom

**Affiliations:** 1 United Nations University International Institute for Global Health (UNU-IIGH), 56000 Cheras, Kuala Lumpur, Malaysia

## Background

The use of quality-adjusted life years (QALY) as an outcome measure of economic evaluations is well-established in the literature. A measure of dental utility analogous to QALY, the quality-adjusted tooth years (QATY), had been initiated but only met with limited success, partly because of its inability to compare across a variety of dental interventions. This study aimed to develop a simple approach to estimate QATY by using an established oral health-related quality of life instrument.

## Materials and methods

Patients newly-diagnosed with periodontitis (n=165, 59% females, mean age 43.3 years) were recruited from five periodontal specialist clinics. They received cause-related periodontal therapy within one year. The Oral Health Impact Profile (OHIP-14) index measured changes in quality-of-life after one year of periodontal treatment. Utility values were derived by converting patients’ OHIP-14 scores of 0-56 to a utility measurement anchored between 0-1, “0” is lowest score corresponding with worst oral health state imaginable, and “1” is highest score with best oral health state imaginable. QATY is calculated by multiplying utility values by tooth life expectancy (TLE).

## Results

Baseline mean utility at baseline was 0.61 (SD ±0.19) and improved to 0.70 (SD ±0.18) after treatment. TLE at baseline was 4.5 years and improved to 30.1 years after treatment. Both improvements were statistically significant (paired t-test, P<0.001). There was significant gain of 21.0 (SD ±7.1) QATY at post-treatment (paired t-test, P<0.001). These improvements in patient-based outcomes were consistent with improvements in clinical parameters: proportions of deep periodontal sites (≥4 mm) decreased from 32.3% to 24.8% (chi-squared test, P<0.001) and gained a median of 0.1 mm (IQR 0.7mm) clinical attachment level (Wilcoxon Signed-Rank test, P<0.001).

## Conclusions

The method of estimating QATY using utilities derived from OHIP-14 combined with tooth life expectancy demonstrated effective treatment outcomes, consistent with improvements in clinical measures. Its use should be considered in economic evaluation of dental treatments.

